# A new species, *Dactylosoma piperis* n. sp. (Apicomplexa, Dactylosomatidae), from the pepper frog *Leptodactylus labyrinthicus* (Anura, Leptodactylidae) from Mato Grosso State, Brazil.

**DOI:** 10.1051/parasite/2020070

**Published:** 2020-12-17

**Authors:** Letícia Pereira Úngari, Edward Charles Netherlands, André Luiz Quagliatto Santos, Edna Paulino de Alcantara, Enzo Emmerich, Reinaldo José da Silva, Lucia Helena O’Dwyer

**Affiliations:** 1 Setor de Parasitologia, DBBVPZ, Instituto de Biociências, Universidade Estadual Paulista-UNESP, Distrito de Rubião Junior Botucatu CEP 18.618-970 São Paulo Brazil; 2 Unit for Environmental Sciences and Management, North-West University Private Bag X6001 Potchefstroom 2520 South Africa; 3 Laboratório de Ensino e Pesquisa em Animais Silvestres, Faculdade de Medicina Veterinária, Universidade Federal de Uberlândia CEP 38.400-902 Minas Gerais Brazil

**Keywords:** Haemoparasite, Haemogregarine, Amphibian, Phylogeny, 18S rRNA

## Abstract

The Dactylosomatidae Jakowska and Negrelli, 1955 are one of four families belonging to adeleorinid coccidia and comprise the genera *Babesiosoma* Jakowska and Nigrelli, 1956 and *Dactylosoma* Labbé, 1894. These blood protozoa occur in peripheral blood of lower vertebrates, and are commonly reported parasitising amphibians. The present study describes *Dactylosoma piperis* n. sp. from the pepper frog *Leptodactylus labyrinthicus* (Spix, 1824) (Anura: Leptodactylidae), collected in 2018 at the municipality of Araguaiana, Mato Grosso State, Brazil, based on morphology of intra-erythrocytic trophozoite, primary and secondary merogonic stages and a molecular analysis (partial 18S rDNA). *Dactylosoma piperis* n. sp. forms a well-supported clade with other Dactylosomatidae. This is the first molecular characterization of a species of *Dactylosoma* from a Brazilian anuran.

## Abstract (fr)

Les Dactylosomatidae Jakowska et Negrelli, 1955 sont l’une des quatre familles appartenant aux coccidies Adeleorina et comprennent les genres *Babesiosoma* Jakowska et Nigrelli, 1956 et *Dactylosoma* Labbé, 1894. Ces protozoaires sanguins se trouvent dans le sang périphérique des vertébrés inférieurs et sont fréquemment signalés comme parasitant des amphibiens. Ce travail décrit *Dactylosoma piperis* n. sp. de la grenouille *Leptodactylus labyrinthicus* (Spix, 1824) (Anura : Leptodactylidae), collectée en 2018 dans la municipalité d’Araguaiana, État du Mato Grosso, Brésil, d’après la morphologie du trophozoïte intra-érythrocytaire, des stades mérogoniques primaires et secondaires et une analyse moléculaire (ADNr 18S partiel). *Dactylosoma piperis* n. sp. forme un clade bien soutenu avec d’autres Dactylosomatidae. Il s’agit de la première caractérisation moléculaire d’une espèce de *Dactylosoma* à partir d’un anoure brésilien.

## Introduction

Haemogregarines (Apicomplexa: Adeleorina) are a diverse group of blood parasites subdivided into four families: Haemogregarinidae Léger, 1911, Hepatozoidae Miller, 1908, Karyolysidae Labbé, 1984, and Dactylosomatidae Jakowska and Nigrelli, 1955. The Dactylosomatidae is a small family that currently comprises the genera *Dactylosoma* Labbé, 1894 and *Babesiosoma* Jakowska and Nigrelli, 1956 [42, 43, 50, 51].

According to Barta [1, 3], members of this family have undergone numerous reclassifications and systematic revisions, since the description of the first species, *Dactylosoma ranarum* (Kruse, 1890). Furthermore, there is a lack of information on the biology of this group of parasites, with the life cycles of only two species elucidated to date, namely *Babesiosoma stableri* Schmittner and McGhee, 1961 and *Babesiosoma mariae* (Hoare, 1930) [4, 6, 64]. Although leeches are considered to be the vectors of these parasites, in a recent study, possible developmental stages of *Dactylosoma kermiti* Netherlands, Cook and Smit, 2020 were observed in the gut and haemocoel of mosquitoes that had fed on infected hosts [64].

Species of *Dactylosoma* are characterised by merogonic development within the peripheral blood of their vertebrate hosts. During primary merogony, a large multinucleate meront is formed producing up to 16 merozoites. These merozoites then separate either repeating primary merogony or initiating secondary merogony. In secondary merogony, meronts produce up to eight merozoites that either repeat secondary merogony or mature into gamonts [65].

Currently there are six recognised species of *Dactylosoma* known globally. Two of these are described from fish hosts, and the remaining four species from anuran hosts. Namely *D. ranarum* described from the European frog *Pelophylax* kl. *esculentus* (Linnaeus, 1758); *Dactylosoma sylvatica* Fanthan, Porter and Richardson, 1942 reported in *Lithobates sylvatica* (LeConte, 1825) from Quebec, Canada; *Dactylosoma taiwanensis* Manwell, 1964 described infecting *Fejervarya limnocharis* Gravenhorst, 1829 collected in Taiwan; and *D. kermiti* described infecting the anurans, *Ptychadena anchietae* Bocage, 1868 and *Sclerophrys gutturalis* Power, 1927 from South Africa. Moreover, to date only two recognised species of *Dactylosoma* have been molecularly characterised, *D. ranarum* and *D. kermiti*, and one unidentified species of *Dactylosoma* from Belgium [65].

In Brazil, only two studies have reported on species of *Dactylosoma* from anuran hosts. Durham [23] briefly reported on two haemogregarine species infecting toads from Para State, the first species an unidentified haemogregarine possessing similar characteristics to *Hemolivia stellata* Petit, Landau, Baccam and Lainson, 1990 and the second a haemogregarine conforming morphologically to a species of *Dactylosoma.* The second was a study by Da Costa and Pereira [21] who screened a total of 100 frogs and toads captured and examined during 1964–1971 from Rio de Janeiro State, Brazil. Parasites from different groups were identified, including species of *Hepatozoon* and *Dactylosoma.* According to Da Costa and Pereira [21], a species of *Dactylosoma* and *Hepatozoon leptodactyli* were observed parasitising *Leptodactylus latrans* (Steffen, 1815) (syn. *L. ocellatus*). These authors suggested that although the dactylosomatid parasite observed resembles *D. ranarum*, more data are needed before final conclusions can be made. To date, there are no formal species descriptions of dactylosomatid species from Brazil.

Due to the limited data of anuran haemogregarine parasites from Brazil, the aim of this study was to characterise and describe a new species of *Dactylosoma* using morphological and molecular methods.

## Materials and methods

### Ethics

All applicable international, national, and institutional guidelines for the ethical handling of animals were followed (IBAMA license 60640-1; CEUA-UNESP 1061).

### Anuran collection

In August of 2018, a female adult of *Leptodactylus labyrinthicus,* with 105.56 mm snout-vent length and weight of 98 g, was collected at the municipality of Araguaiana, Mato Grosso State, Brazil (14°35′47″ S; 51°43′9.59″ W) (FAPESP 2018/09623-4; FAPESP 2018/00754-9). The animal was physically restrained and blood was collected by puncture of the cervical paravertebral sinus using sterile and disposable syringes and needles [81]. During the containment, the sex (male/female) and age of the specimen were estimated. No ectoparasites were observed on the animal.

After the blood collection, three thin blood smears were made on glass slides and the remaining blood sample was stored in EDTA tubes and frozen at −10 °C for further molecular analysis.

### Morphological and morphometric analysis

The blood smears were fixed with absolute methanol and stained with 10% Giemsa Methylene Blue Eosin Merck^®^ diluted in distilled water (pH 7.0 for 50 min), according to Eisen and Schall [26], at the Parasitology division from UNESP, Botucatu. For morphological analysis of the intra-erythrocytic parasite stages, digital images were captured and measured using a compound microscope at 1000× magnification with the Leica software application suite LAS V3.8 (Leica Microsystems). Measurements are in micrometres (μm) comprising the parasite’s length and width, with mean and standard deviation (means ± standard deviation) given. Parasitaemia was calculated per 100 erythrocytes, with ~10^4^ erythrocytes examined per blood smear following Cook et al. [16].

### Molecular analysis

DNA was extracted from whole blood samples following the blood protocol of the DNeasy Blood and Tissue Kit (Qiagen, Valencia, CA, USA). Partial 18S rRNA gene fragments (600 bp) were amplified using the primers HepF300/Hep900 [79]. PCR amplification reactions were carried out in a final volume of 25 μL, containing 1 μL each of 10 pmol primers, 12.5 μL of Master Mix MyFi^TM^ Mix Bioline^®^ and 5 μL of extracted DNA, with nuclease-free water accounting for the remaining volume; following the conditions of O’Dwyer et al. [69]. PCR amplification was performed on a Peltier 200 Thermocycler (MJ Research, Watertown, MA, USA), with initial denaturation at 94 °C for 3 min, followed by 35 cycles of 94 °C for 45 s, 50 °C for 60 s and 72 °C for 60 s, followed by a final extension at 72 °C for 7 min.

PCR products were subjected to electrophoresis at 80 V in a 1.5% agarose gel, stained with Gel Red, and observed using ultraviolet transilluminator. The products of interest were purified by adding 2 μL of ExoSAP-IT^®^ (Affymetrix, Santa Clara, CA, USA) to 5 μL of PCR product according to the manufacturer’s recommendations. Amplicons were then sequenced using PCR primers on a 3500 Genetic Analyzer capillary sequencer (Applied Biosystems) and after using a BigDye Terminator Cycle Sequencing Ready Reaction Kit v.3.1 (Applied Biosystems), according to the manufacturer’s recommendations.

The sequence chromatograms obtained (forward and reverse sequences) were assembled and edited using BioEdit v.7.0.9 [34] to obtain a partial 18S rDNA consensus sequence. Sequences from the haemogregarine group were aligned using Geneious version 7.1.3 [46] with the MUSCLE algorithm (Bomatters, www.geneious.comww). *Adelina dimidiata* Schneider, 1875, *Adelina grylli* Butaeva, 1996, *Klossia helicina* Schneider, 1875 and *Klossiella equi* Baumann, 1945 from the suborder Adeleorina were selected as outgroups following Netherlands et al. [64]. Alignment gaps and ambiguities were removed using the Gblocks server [12, 76]. JModelTest v.2.1.10 [20] was used to determine the most suitable nucleotide substitution model. Based on the Akaike information criterion (AIC) the General Time Reversible [77] model with estimates of invariable sites and a discrete Gamma distribution (GTR + I + Γ) was selected as the best model. Phylogenetic relationships were inferred via Bayesian inference (BI) using MRBAYES 3.2.2 [40] and Maximum likelihood (ML) analysis using RAxML 7.2.8. [32, 76], implemented in Geneious R7. For the BI analysis, the Markov Chain Monte Carlo (MCMC) algorithm was run for 1 million generations, sampling every 100 generations. The first 25% of the trees were discarded as “burn-in”. The Tracer tool was used to assess convergence and the “burn-in” period [71]. For the ML analysis, nodal support was assessed using 1000 rapid bootstrap replicates [72]. The aligned sequences of *Dactylosoma* species from anurans were compared using a pair-wise distance (p-distance) matrix.

## *Dactylosoma piperis* Úngari, Netherlands, Silva & O’Dwyer n. sp.

urn:lsid:zoobank.org:act:92749FA8-8673-4556-B03F-F925A15B8A07

*Type-host: Leptodactylus labyrinthicus* (Anura: Leptodactylidae).

*Type-locality:* Municipality of Araguaiana, Mato Grosso State, Brazil (coordinates 14°35′47″ S 51°43′9.59″ W).

*Site of infection:* Peripheral blood erythrocytes.

*Parasitaemia:* 0.2%.

*Etymology*: The host species *L. labyrinthicus* is commonly referred to in Brazil as the pepper frog. Therefore, the species epithet is derived from the Latin word *piperis* meaning pepper (noun in apposition).

*Material deposited:* Hapantotype, two blood smears from *L. labyrinthicus* deposited in the collection of the National Institute of Amazonian Research (INPA), Manaus, Brazil [INPA19a, INPA19b].

*Gene sequence:* 18S rRNA gene sequence deposited in GenBank under accession number MW264134.

*Note:* The authors of the new taxon are different from the authors of this paper; Article 50.1 and Recommendation 50A of the International Code of Zoological Nomenclature [41].

### Description (Fig. 1; Table 1):

**Figure 1:**
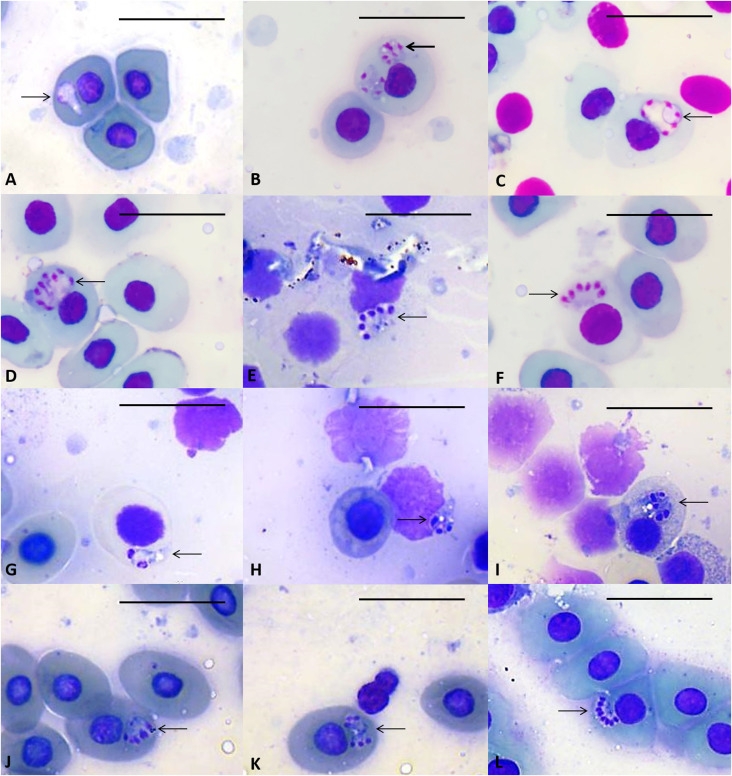
*Dactylosoma piperis* n. sp. in blood smears of *Leptodactylus labyrinthicus*. Primary merogony (A–F): A) Trophozoite; B) Young primary meront; C–D) Primary large rounded meronts; E–F) Fan-like shaped primary meronts with merozoites. Secondary merogony (G–L): G–H) Young secondary meronts; I–J) Secondary meronts with dactylate appearance; K–L) Secondary meronts with merozoites. Scale bar: 10 μm.

The developmental stages of the unidentified species of *Dactylosoma* observed were trophozoites, early stage meronts, meronts and merozoites from the primary merogony. For secondary merogony, it was possible to identify early stage meronts, meronts and merozoites. In addition, the early stage meronts and the mature meronts varied in morphology including the typical hand-like (dactylate shape), the quadrangular, the fan-like and circular shapes. Typically, primary merogony of species of *Dactylosoma* produces up to 16 merozoites and secondary merogony up to eight merozoites. However, in the present study, during primary merogony, meronts were observed producing up to ten chromatin divisions of the nuclei and during secondary merogony, meronts were observed producing up to eight chromatin divisions (Fig. 1).

**Table 1:** Morphometric data on developmental stages of validated *Dactylosoma* species from fishes and anuran hosts around the world.

			Morphometric data (μm)				
Species	Host(s)	Country	Trophozoites (μm)	Meronts – M (μm)	Merozoites – Me (μm)	Gametocytes (μm)	Reference
	**Fish**						
*Dactylosoma salvelini* Fantham, Porter and Richardson, 1942	*Salvelinus fontinalis* Mitchill, 1814	Canada	N/A	2nd M: 5.8–8.5 × 3.7–7.0	N/A	4.4–7.8 × 1.5–3.0	[27]
*Dactylosoma lethrinorum* Saunders, 1960	*Lethrinus nebulous* Forsskål, 1775*; L. lentjan* Lacepède, 1802	Egypt	N/A	1st. M: 8.0 × 10.5	1st. M: 1.9 × 2.4	N/A	[73]
	**Anuran**						
*Dactylosoma sylvatica* Fantham, Porter and Richardson, 1942	*Lithobates sylvatica* LeConte, 1825	Canada	1st. M: 7.0–8.5 × 6.3–7.6	1st. M: 7.4–11.5 × 7.0–9.3		7.0–12.6 × 1.5–3.0	[27]
			2nd M: 4.4 × 3.0	2nd M: 5.2 × 4.0	2nd M: 4.4–5.9 × 1.1–2.0		
*Dactylosoma taiwanensis* Manwell, 1964	*Fejervarya limnocharis* Gravenhorst, 1829	Taiwan	1st. M: 3.9 × 7.3	2nd. M: 6.9–7.9 × 5.6–7.3	N/A	11.8–13.6 × 2.1–2.9	[60]
*Dactylosoma ranarum* Kruse, 1890 (syn*. D. splendes)*	*Pelophylax* kl. *esculentus* Linnaeus, 1758		1st. M: 3.0–4.0 × 1.5–2.0	1st. M: 10.0–15.0 × 2.0–3.0; 7.3 × 4.3	1st. M: 2.8 × 0.7; 4.3 × 1.3	5.0–8.0 × 1.5–3.0; 7.0 × 3.4	[5, 48]
				2nd. M: 9.0 × 4.0; 4.7 × 3.4	2nd. M: 2.0–3.0 × 1.0–1.5; 3.4 × 0.9		
*Dactylosoma kermiti* Netherlands et al., 2020	*Ptychadena anchietae* Bocage, 1868; *Sclerophrys gutturalis* Power, 1927	South Africa	1st. M: 5.3–7.7 × 2.6–4.4	1st. M: 8.3–12.2 × 5.1–8.0	1st. M: 5.0–6.6 × 1.8–3.2	7.8–15.0 × 1.5–3.0	[65]
				2nd. M: 5.6–8.6 × 4.4–6.9	2nd. M: 4.2–5.5 × 1.8–3.5		

#### Primary merogony

*Trophozoite* (Fig. 1A): Elongated, tapering towards one end and larger and rounded at opposite end, measuring 7.4 μm ± 1.3 μm long, 3.75 μm ± 1.5 μm wide, and with area of 19.31 μm^2^ ± 0.4 μm^2^; cytoplasmic vacuoles observed mainly in tapering end; nuclei placed at the rounded end, although chromatin division is not clearly defined; cytoplasm staining whitish-purple (*n* = 5).

*Young primary meronts* (Fig. 1B): Ovoid to round shape with dispersed vacuoles, measuring 5.20 μm ± 0.15 μm in length, 5.53 μm ± 0.7 μm in width, with area of 20.41 μm^2^ ± 0.4 μm^2^; multinucleate, with between four to six nuclei located peripherally and staining purple; causes displacement of host nuclei and cell (*n* = 2),

*Primary meronts* (Figs. 1C–1D): Large rounded meronts, measuring 8.59 μm ± 0.2 μm in length, 6.73 μm ± 0.5 μm in width, with area of 31.40 μm^2^ ± 0.4 μm^2^; causing slight distortion and displacement of host cell nucleus; multinucleate with between 6 and 10 nuclei located peripherally; purplish or pinkish staining chromatin (*n* = 3).

*Primary meronts with merozoites* (Figs. 1E–1F): Large fan-shaped meronts with distinct triangular form, measuring 8.38 μm ± 0.1 μm in length, 6.71 μm ± 0.25 μm in width, with area of 31.24 μm^2^ ± 0.5 μm^2^ (*n* = 3); multinucleate with ovoid dense chromatin positioned on one side of the parasite, usually displacing erythrocyte nuclei, chromatin staining dark purple or pinkish; merozoites measurements 7.45 μm ± 0.25 μm in length and 2.90 μm ± 0.25 μm in width (*n* = 30).

#### Secondary merogony

*Young secondary meront* (Figs. 1G–1H): Elongated with one end tapered and the other rounded. Rounded end containing two to three nuclei, with dense and circular chromatin staining in deep magenta peripherally distributed, with or without cytoplasmic vacuole, 6.1 μm ± 1.2 μm length, 4.15 μm ± 0.9 μm width and 28.02 μm^2^ ± 0.2 μm^2^ in area (*n* = 3).

*Secondary meronts* (Figs. 1I–1J): Dactylate (hand-like) appearance, ovoid to round shape, 6.9 μm ± 0.4 μm length, 5.6 μm ± 0.2 μm width and 25.53 μm^2^ in area (*n* = 2); multinucleate with between five and eight nuclei located peripherally with dense chromatin staining in deep magenta.

*Secondary meronts with merozoites* (Figs. 1K–1L): Morphology varying from fan-like shape to quadrangular shape. Multinucleate with between six and eight nuclei with chromatin division located peripherally, with or without vacuoles; in some cases, slight displacement of host cell nucleus evident. Quadrangular shape meront (Fig. 1K): Multinucleate with six rounded nuclei, three dense nuclei positioned on each side of the meront, forming a square-shape, measuring 7.54 μm ± 0.2 μm long, 5.4 μm ± 0.9 μm wide, and with area of 25.88 μm^2^ (*n* = 2). Merozoites measured 6.2 μm ± 0.2 μm long and 1.5 μm ± 0.9 μm wide (*n* = 12). Fan-like shape meront (Fig. 1L): Multinucleate with 8 nuclei, ovoid dense chromatin positioned on one side of meront, forming fan-like shape; usually displacing host cell nucleus, measuring 6.95 μm long, 4.89 μm wide, and with area of 23.68 μm^2^ (*n* = 1). Merozoites measured 5.88 μm ± 0.2 μm long and 1.3 μm ± 0.9 μm wide (*n* = 8).

### Differential diagnosis

*Dactylosoma piperis* n. sp. is characterised by its elongated and unique trophozoites, with one side rounded and the other tapered; the morphological variation of early stage meronts to mature meronts ranging between dactylate, fan-like, quadrangular and circular shapes, and the number of merozoites produced in primary merogony (up to 10) and secondary merogony (up to eight).

This species can be distinguished from all currently recognised species of *Dactylosoma* from anuran hosts, namely *D. kermiti*, *D. ranarum*, *D. sylvatica*, and *D. taiwanensis* based on several developmental characteristics, such as the number of nuclear chromatin divisions present in primary and secondary merogony, unique trophozoite morphology and developmental stage morphometrics.

In comparison, *D. piperis* n. sp. differs from *D. ranarum* (the first described species in the Dactylosomatidae), in the number of chromatin divisions of up to six nuclei during secondary merogony and trophozoite morphology being slender and smaller with both ends rounded. Nevertheless, certain characteristics observed, such as meronts with merozoites arranged in fan-like fashion or quadrangular mass, and the two types of schizogony (primary and secondary), are typical of dactylosomatid parasites. The first type producing larger meronts with nuclei located peripherally and vacuoles present during merozoite formation, and the second type producing smaller meronts, with chromatin division of nuclei more condensed and staining dark-purple with fewer merozoites produced.

For *D. kermiti*, primary merogony is characterised by up to 14 chromatin divisions and second merogony by up to six chromatin divisions, as compared to *D. piperis* n. sp., with up to 10 and up to 8 chromatin divisions observed in primary and secondary merogony, respectively. In addition, the trophozoites of *D. kermiti* are smaller and slender, and elongated to oval in shape with vacuoles present, measuring 6.7 μm ± 2.2 μm long and 3.5 μm ± 1.2 μm wide, which differs from the trophozoite size and shape of *D. piperis* n. sp. Also, the morphometric values of primary meronts and merozoites, and secondary meronts and merozoites from this study were larger as compared to *D. kermiti*.

In comparison, the developmental stages between *D. sylvatica* and *D. piperis* n. sp. differ in morphology and size, with trophozoites of *D. sylvatica* measuring lager (7.0–8.5 μm × 6.3–7.6 μm), with an amoeboid shape, circular nuclei and alveolar cytoplasm without inclusions. Furthermore, meronts of *D. sylvatica* produce only up to eight merozoites, in the first and the second merogony, as compared to up to ten in the first merogony and eight in the second merogony of *D. piperis* n. sp.

With regard to *D. taiwanensis* and *D. piperis* n. sp., both species present similar trophozoite morphology, with trophozoites of *D. taiwanensis* measuring 3.9 μm wide and 7.3 μm long; distinguished morphology can be observed, with elongate or ovoid vacuolated form with equal rounded ends, compared to elongated with one end rounded and the other tapered from *D. piperis* n. sp. From secondary merogony, mature fan-like, quadrilateral-shape and hand-like meronts were observed with between four and eight nuclei, differing from the mature meronts of *D. piperis* n. sp. with between six and eight nuclei.

### Molecular and phylogenetic analysis (Fig. 2; Tables 2–3)

**Figure 2:**
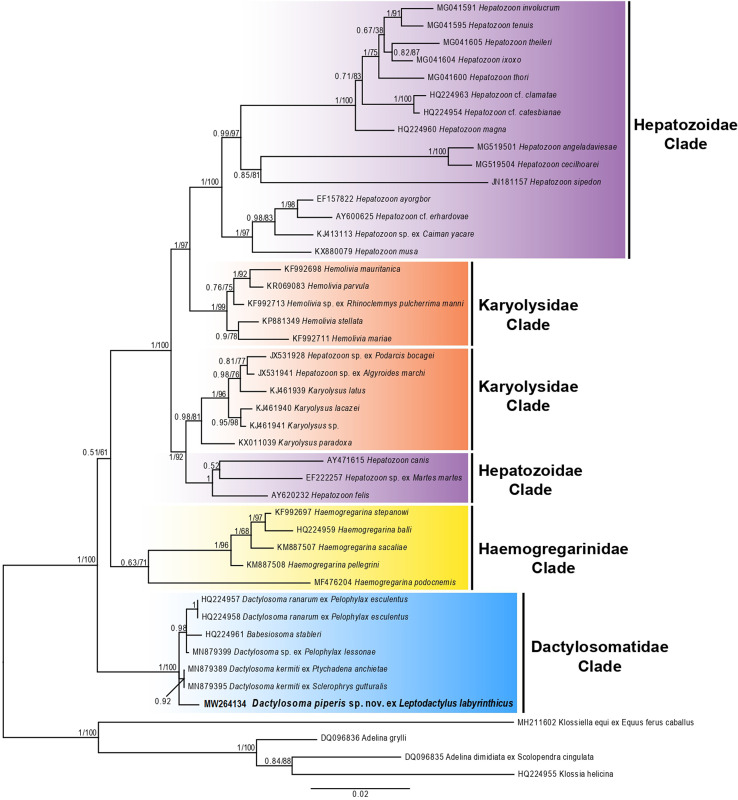
Consensus phylogram of haemogregarines based on 18S rDNA sequences. The topology trees with Bayesian inference (BI) and Maximum likelihood (ML) analyses were identical (represented by the ML tree). The scale bar represents 0.02 nucleotide substitutions per site. *Adelina dimidiata* (DQ096835), *Adelina grylli* (DQ096836), *Klossia helicina* (HQ224955) and *Klossia equi* (MH211602) were used as out-groups.

The phylogenetic tree comprised sequences of adeleorinid apicomplexan parasites (Haemogregarinidae, Hepatozoidae, Karyolysidae, and Dactylosomatidae) available from GenBank (Table 2). The BI and ML phylogenetic analysis had similar topologies, showing species of *Haemogregarina* forming a monophyly sister to a large clade consisting of isolates from species of *Hepatozoon*, *Hemolivia*, and *Karyolysus*. All species of *Dactylosoma* clustered together as a sister group to the Haemogregarinidae clade (Fig. 2).

**Table 2:** GenBank accession numbers, hosts, country and citation for SSU rDNA sequences of haemogregarines from reptiles, amphibians and mammal used in the phylogenetic analyses (except the sequence from this study).

Species	GenBank Number	Host	Country	Citation
*Dactylosoma kermiti* Netherlands, Cook and Smit, 2020	MN879392	*Ptychadena anchietae* Bocage, 1868	South Africa	[65]
*Dactylosoma kermiti*	MN879398	*Sclerophrys gutturalis* Power, 1927	South Africa	[65]
*Dactylosoma ranarum* Kruse, 1890	HQ224958	*Rana esculenta* Linnaeus, 1758	Canada	[1]
*Dactylosoma ranarum*	HQ224957	*Rana esculenta*	Canada	[1]
*Dactylosoma* sp.	MN879399	*Pelophylax lessonae* Camerano, 1882	Belgium	[65]
*Babesiosoma stableri* Schmittner and Mc Ghee, 1961	HQ224961	*Rana septentrionalis* Baird, 1854	Canada	[1]
*Haemogregarina podocnemis* Úngari, Santos, O′Dwyer, Silva, Fava, Paiva and Cury, 2018	MF476204	*Podocnemis unifilis* Troschel, 1848	Brazil	[80]
*Haemogregarina balli* Peterson and Desser, 1976	HQ224959	*Chelydra serpentina serpentina* Linnaeus, 1758	Canada	[1]
*Haemogregarina sacaliae* Devořáková, 2015	KM887507	*Sacalia quadriocellata* Siebenrock, 1903	Vietnam	[24]
*Haemogregarina pellegrini* Laveran and Petit, 1910	KM887508	*Malayemys subtrijuga* (Schelegel and Mũller, 1845)	Vietnam	[24]
*Haemogregarina stepanowi* Danilewsky, 1885	KF992697	*Mauremys caspica* (Gmelin, 1774)	Turkey	[25]
*Hemolivia stellata* Petit, Landau, Baccam and Lainson, 1990	KP881349	*Amblyomma rotundatum* Kock, 1844	Brazil	[43]
*Hemolivia mauritanica* Petit, Landau, Baccam and Lainson, 1990	KF992698	*Testudo graeca* Linnaeus, 1758	Turkey	[49]
*Hemolivia parvula* Dias, 1953	KR069083	*Kinixys zombensis* Hewitt, 1931	South Africa	[17]
*Hemolivia mariae* Smallridge and Paperna, 1997	KF992711	*Egernia stokesii* (Gray, 1845)	Australia	[49]
*Hemolivia* sp.	KF992713	*Rhinoclemmys pulcherrima manni* (Dunn, 1930)	Nicaragua	[49]
*Hepatozoon cf. catesbianae* (Stebbins, 1903) Desser, Hong and Martin, 1995	HQ224954	*Lithobates catesbeianus* (Shoaw, 1802) Dubois, 2006	Canada	[1]
*Hepatozoon ixoxo* Netherlands, Cook and Smit, 2014	KP119772	*Amietophrynus maculatus* Hallowell, 1854	South Africa	[67]
*Hepatozoon theileri* Laveran, 1905	KJ461939	*Amietia quecketti* Boulenger, 1895	South Africa	[67]
*Karyolysus paradoxa* (Dias, 1954) Cook, Netherlands and Smit, 2016	KX011039	*Varanus albigularis* Daudin, 1802	South Africa	[16]
*Karyolysus lacazei* Zechmeisterova, Bellocq and Siroky, 2019	KJ461940	*Lacerta agilis* Linnaeus, 1758	Poland	[33]
*Karyolysus latus* Haklová-Ko, 2014	KJ461939	*Podarcis muralis* Laurenti, 1768	Slovakia	[33]
*Karyolysus* sp.	KJ461939	*Lacerta viridis* Laurenti, 1768	Hungary	[49]
*Hepatozoon felis* Patton, 1908	AY620232	*Felis catus* Linnaeus, 1758	Spain	[14]
*Hepatozoon* sp.	EF222257	*Martes martes* Linnaeus, 1758	Spain	[13]
*Hepatozoon canis* Christophers, 1907	AY471615	*Pseudalopex gymnocercus* Fischer, 1814	Brazil	[14]
*Hepatozoon involucrum* Netherlands, Cook and Smit, 2017	MG041591	*Hyperolius marmoratus* Rapp, 1842	South Africa	[66]
*Hepatozoon tenuis* Netherlands, Cook and Smit, 2017	MG041595	*Afrixalus fornasini* (Bianconi, 1849)	South Africa	[66]
*Hepatozoon thori* Netherlands, Cook and Smit, 2017	MG041600	*Hyperolius marmoratus* Rapp, 1842	South Africa	[66]
*Hepatozoon cf. clamatae* (Stebbins, 1905) Smith, 1996	HQ224963	*Lithobates clamitans* (Latreille, 1801)	Canada	[1]
*Hepatozoon magna* (Grassi and Felletti, 1891) Labbé, 1899	HQ224960	*Pelophylax esculentus* Linnaeus, 1758	Canada	[1]
*Hepatozoon angeladaviesae* Cook, Netherlands, Van As and Smith, 2018	MG519501	*Philothamnus hoplogaster* Bocage, 1882	South Africa	[13]
*Hepatozoon cecilhoarei* Cook, Netherlands, Van As and Smith, 2018	MG519504	*Philothamnus natalensis natalensis* (Smith, 1848)	South Africa	[13]
*Hepatozoon sidepon* Smith, Desser and Martin, 1994	JN181157	*Nerodia sipedon sipedon* Linnaeus, 1758	Canada	[1]
*Hepatozoon ayorgbor* Sloboda, Kamler, Bulantova, Votypka and Modry, 2007	EF157822	*Phyton regius* Shaw, 1802	Ghana	[75]
*Hepatozoon cf. erhardovae* Criado-Fornelio, 2006	AY600625	*Clethrionomys glareolus* Schreber, 1780	Spain	[14]
*Hepatozoon* sp.	KJ413113	*Caiman yacare* Daudin, 1802	Brazil	[8]
*Hepatozoon musa* Borges-Nojosa, Borges-Leite, Maia, Zanchi-Silva, Braga and Harris, 2017	KX880079	*Phylodryas nattereri* Steindachner, 1870	Brazil	[7]
*Hepatozoon* sp.	JX531928	*Podarcis bocagei* (Lopez-Seoane, 1885)	Portugal	[57]
*Hepatozoon* sp.	JX531941	*Algyroides marchi* Valverde, 1958	Portugal	[57]
*Adelina dimidiata* Schneider, 1875	DQ096835	*Scolopendra cingulate* Latreille, 1829	Bulgaria	[47]
*Adelina grylli* Butaeva, 1996	DQ096836	*Gryllus bimaculatus* De Geer, 1773	Bulgaria	[47]
*Klossia helicina* Schneider, 1875	HQ224955	*Cepaea nemoralis* (Linnaeus, 1758)	France	[1]
*Klossiella equi* Baumann, 1945	MH211602	*Equus ferus caballus* Boddaert, 1785	Canada	[56]

**Table 3:** The shaded matrix (upper) shows the percentage of similarity (%) of the nucleotide sequences and the non-shaded matrix (lower) shows the p-distance (pair-wise distance) between the *Dactylosoma* sequences in anurans available at GenBank (452 nt).

	1	2	3	4	5	6
1. *Dactylosoma piperis* n. sp. (MW264134)		99.27	99.27	98.91	98.91	98.91
2. *Dactylosoma kermiti* (MN879398)	0.005		100	99.45	99.45	99.45
3. *Dactylosoma kermiti* (MN879392)	0.005	0.000		99.45	99.45	99.45
4. *Dactylosoma* sp. (MN879399)	0.009	0.005	0.005		100	100
5. *Dactylosoma ranarum* (HQ224957)	0.009	0.005	0.005	0.000		100
6. *Dactylosoma ranarum* (HQ224958)	0.009	0.005	0.005	0.000	0.000	

*Dactylosoma piperis* n. sp. (MW264134) is well nested within the Dactylosomatidae clade, forming a sister taxon to *D. kermiti* (MN879398/MN879392). Moreover, the genetic distances of the isolate from this study and dactylosomatid sequences available on GenBank showed interspecific divergence of 0.63% with *D. kermiti* (MN879398/MN879392) and 1.90% with *D. ranarum* (HQ224957/HQ224958), and the pair-wise distance varied from 0.005 to 0.009 (452 nt) (Table 3).

## Discussion

Amphibians are experiencing large-scale declines in species diversity. According to the IUCN Global Amphibian Assessment over the past decade, a third of the estimated amphibian species have declined. The major contributors to amphibian’s species declines are environmental changes, fragmentation, and loss of habitat [29, 30]. In addition, this group of vertebrates has a great diversity of parasites, ranging from helminths, bacteria and fungi to haemoparasites, such as trypanosomatids and haemogregarines [2, 27, 39, 54, 67, 68]. Moreover, one disease has recently caught the attention of the scientific community: the amphibian chytridiomycosis panzootic is considered the most impactful example of disease spread and demonstrates its role in the decline of amphibian biodiversity worldwide [74]. However, although parasites usually have a negative connotation, they play a fundamental role in biology, ecology, evolution and population dynamics [39].

Costa and Bérnils [19] reported that Brazil has the greatest biodiversity of amphibians in the world, with more than 1,080 described species. Yet, studies on amphibian parasites from Brazil are scarce especially with regards to protozoan haemoparasites, such as the haemogregarines [21, 23]. Therefore, there is a lack of data on the diversity, life cycles and possible vectors of protozoan haemoparasites of Brazilian anurans, highlighting the importance of screening these diverse hosts in Brazil [2, 31].

The *L. labyrinthicus* was infected by a species of *Dactylosoma*. This anuran is widely distributed throughout South America [29, 36, 37]. In Brazil, *L. labyrinthicus* occurs mainly near wetlands and has been recorded in open habitats throughout the Cerrado, Caatinga regions, and in central Amazonia [11, 38, 52, 57]. It is a large frog from the *Leptodactylus* group [35] and opportunist predator feeding on invertebrate and vertebrate animals (amphibians, amphisbaenas, lizards, snakes, and small rodent species) [10, 28, 78, 81]. In the IUCN Red list, this species is classified as LC – Least Concern [35].

In the present study from the blood smears of *L. labyrinthicus*, a new species of *Dactylosoma*, *Dactylosoma piperis* n. sp. is described, with parasitaemia of 0.2%. In a recent study, Netherlands et al. [65] described *D. kermiti* infecting anurans in South Africa, with parasitaemia varying between host species and individuals. In the host *Ptychadena anchietae* (Bocage, 1868), parasitaemia varied from 2% to 5.7%, and in the host *Sclerophrys gutturalis* (Power, 1927), parasitaemia averaged 0.2%, similar to the current study’s findings.

In Brazil, studies of haemogregarine prevalence and parasitaemia from anurans are scarce. Da Sousa and Filho [22] reported 1% prevalence of *Haemogregarina* from 100 anurans screened. Intra-erythrocytic gamonts infecting the blood smears of one *Rhinella crucifer* (Wied-Neuwied, 1821) (syn. *Bufo crucifer*) from Rio de Janeiro State, Brazil, were found with parasitaemia 0.5%.

In another study by Kattar [45], from 100 Brazilian anurans analysed, eight (8%) were positive for haemogregarine parasites infecting blood smears of *Rhinella diptycha* (Cope, 1862) (syn. *Bufo paracnemis*) collected at João Pessoa City, Paraíba State, Brazil. However, gametocyte morphology was similar to that of the genus *Hemolivia*.

Using microscopy screening of blood smears, Leal et al. [54] reported a 10% prevalence of haemogregarines in the Brazilian frogs *Leptodactylus chaquensis* Cei, 1950, *L. podicipinus* Cope, 1862 and *Phyllomedusa hypocondrialis* Daubin, 1800, from Mato Grosso do Sul State and São Paulo State.

Regarding species of *Dactylosoma*, Da Costa and Pereira [21] observed a species of *Dactylosoma* infecting *L. latrans* (Steffen, 1815) (syn. *L. ocellatus*) from Rio de Janeiro State with low prevalence reported only in the fall and winter season (1964–1971); however, no morphometric data are available for these observations. The only developmental stages reported were meronts conforming to secondary merogonic early meronts with nuclei located at the rounded periphery of the parasite, and a fan-like shaped meront with four nuclei.

Species of *Dactylosoma* have a wide distribution, infecting a variety of hosts [9, 53, 60]. These findings support the hypothesis of parasite distribution proposed by Metcalf [62], suggesting that parasite distribution could be explained by a Gondwana land link, so the same species could be reported in different hosts from distant geographic regions; however, according to Manwell [60], this theory was never accepted. However, the geographical locations of the six valid species do not include the regions of Central- and South America. Therefore, it is unlikely that *D. piperis* n. sp., is a previously described species from a different continent, with different biomes, ecosystems and also different vertebrate hosts and possible vectors. All these data support the description of *D. piperis* n. sp. as a new species with the aid of morphological and molecular analysis.

With regard to the molecular analysis, the phylogenetic relationships between different haemogregarines (Karyolysidae, Haemogregarinidae, Hepatozoidae, and Dactylosomatidae) and the isolate from the present study showed the forming of several well-supported clades. Species of *Hepatozoon* were polyphyletic, with species isolated from large mammals forming a well-supported clade sister to the Karyolysidae clade comprising species of *Karyolysus*, with species of *Hepatozoon* isolated from amphibians, reptiles and rodents forming a well-supported clade sister to the Karyolysidae clade comprising species of *Hemolivia*. The Haemogregarinidae clade formed a sister clade to the large monophyly comprising the Hepatozoidae and Karyolysidae clades. The Dactylosomatidae clade was found to be a well-supported monophyletic group sister to the Haemogregarinidae clade, these findings are similar to those reported by Netherlands et al. [65]

In addition, the Dactylosomatidae clade formed a polytomy with *Babesiosoma stableri* (HQ224961); *Dactylosoma* sp. (MN879399) and *D. ranarum* (HQ224957/HQ224958) formed a monophylum; and *D. kermiti* (MN879398/MN879392) and *D. piperis* n. sp. (MW264134) nested within the polytomy. Despite the low interspecific divergence (*p*-distance 0.005–0.009) between dactylosomatid species, the 18S gene distinguished *D. piperis* n. sp. as a separate species. Thus, although the 18S rRNA gene is a conservative marker, it has provided stability between closely related genera and species within Adeleorina [15, 18, 59, 61, 68, 70].

Moreover, in an attempt to resolve the polyphyletic genus *Hepatozoon*, the genus *Bartazoon* Karadjian, Chavatte and Landau, 2015, was proposed to replace the *Hepatozoon* genus from species transmitted exclusively by haematophagous insects, with the aim of resolving the polyphyletic placement of the genus *Hepatozoon* [44]. However, Maia et al. [58] considered the idea premature, since some issues within *Hepatozoon* polyphyly still remain unsolved even with the use of the proposed genus *Bartazoon*.

Furthermore, Léveillé et al [55], shows the problematic designation of the genus *Bartazoon* based on the congenic and phylogenetic relationship of *Hepatozoon griseisciuri* and the type species of the genus *Hepatozoon* as described by Miller [63]. Léveillé et al. [55] reporting complete molecular data on nuclear 18S rDNA and the mitochondrial genome from *Hepatozoon* spp., showed significant pairwise differences observed between 18S rDNA and mitochondrial genome sequences; the sequences observed in their study support the idea of superiority of COI sequences on nuclear genes to describe species, and the mitochondrial genomes sequenced to date display staggering diversity. The adeleorinid coccidian will require additional sequence data from mitochondrial genomes to better understand the taxonomy and phylogenetic classifications, and the authors suggested that the genus *Hepatozoon* is likely distributed into multiple genera that have yet to be defined.

Notwithstanding, until the scientific community has complete knowledge about the transmission structure and life cycle of haemogregarines, as well as the real phylogenetic and genomic diversity, the addition of a new genus to the group is precipitated. Thus, the phylogenetic analysis from this study was based on Léveillé et al. [55] and Maia et al. [58], within the old classification of *Hepatozoon* spp., covering different groups of animals and transmission pathways, considered valid so far.

The importance of using techniques to correctly identify and describe a new species is emphasized in this manuscript. However, regarding the molecular technique, future studies should include using variable markers, such as a mitochondrial gene, to increase the phylogenetic resolution and systematic position on dactylosomatid parasites. Furthermore, studies including a great variety of Brazilian anuran species from different localities should be done with the aim of increasing the biodiversity and prevalence knowledge of dactylosomatid species. Also, studies focusing on life-cycle experimental work, testing possible vectors in the transmission *D. piperis* n. sp., should be attempted to gain a better understanding of the ecology of this parasite.

This study provides the first report with molecular characterisation of a species of *Dactylosoma* parasitising Brazilian anurans.

## Conflict of interest

The authors declare that they have no conflict of interest.

## Funding

R.J.S. is supported by FAPESP (2016/50377-1), CNPq (309125/2017-0), and CNPq-PROTAX (440496/2015-2). L.P.U is supported by FAPESP (2018/00754-9; 2018/09623-4). L.H.O is supported by FAPESP (2018/09623-4).
